# Sampling criteria for identifying human biomonitoring chemical differences in the Canadian Arctic

**DOI:** 10.3402/ijch.v73.23467

**Published:** 2014-02-17

**Authors:** Meredith S. Curren, Karelyn Davis, Jay Van Oostdam

**Affiliations:** 1 Chemicals Surveillance Bureau, Healthy Environments and Consumer Safety Branch, Health Canada, Ottawa, Ontario, Canada; 2 Environmental Health Science and Research Bureau, Healthy Environments and Consumer Safety Branch, Health Canada, Ottawa, Ontario, Canada; 3 Consultant, Ottawa, Ontario, Canada

**Keywords:** Arctic, Inuit, human biomonitoring, persistent organic pollutants, mercury, Canada

## Abstract

Human biomonitoring studies in the Canadian Arctic have measured a wide range of metals and persistent organic pollutants in Aboriginal and non-Aboriginal mothers during two time periods in the Northwest Territories and Nunavut. This analysis provides preliminary estimates on sample sizes and sampling frequencies required to measure significant changes in maternal blood concentrations for PCB 153 and total mercury. For example, sample sizes of 35–40 mothers permit the detection of a 40% decrease in these chemical concentrations between two groups (e.g. communities or regions). Improvements in method sensitivity can be achieved by on-going sampling over multiple time periods (e.g. 4 or 5) in these regions, or increasing sample sizes.

Persistent organic pollutants (POPs) and mercury biomagnify in northern traditional foods and have been linked to human health effects, with most concern placed on the immune, nervous, hormonal or cardiovascular systems of the foetus. Accordingly, human biomonitoring studies conducted in the Canadian Arctic have frequently examined expectant women to obtain insight on potential risks to the developing child.

Northern studies indicate that concentrations of many historic POPs have declined in Arctic biota (1) and Canadian Arctic peoples (2) over the past few decades. The fate of mercury in the Arctic is less clear, as both increasing and decreasing concentrations have been observed in biota (3), while human concentrations in Canada have shown a general decline (2). In view of this ambiguity, it becomes desirable to characterize temporal and spatial trends using clear quantitative measures. A statistically robust regression-based analysis method has been developed to describe temporal trends of POPs (1) and mercury (3) in Arctic biota. Although this approach has been undertaken for the general Inuit population in Greenland (4), to our knowledge this kind of statistical rigor has not been used to identify chemical trends or define sampling criteria for human biomonitoring in the Canadian Arctic. To inform future study design, we performed a preliminary statistical analysis of two previous northern biomonitoring studies to estimate sample sizes and sampling frequencies required to report significant changes in blood concentrations for PCB 153 and mercury in mothers from the Northwest Territories and Nunavut.

## Methods

### Study population

The Northern Contaminants Program (NCP) of Aboriginal Affairs and Northern Development Canada has coordinated maternal biomonitoring studies in the Inuvik Region of the Northwest Territories and the Baffin Region of Nunavut on two occasions (1997–1999 and 2005–2007) (2). All expectant Aboriginal and non-Aboriginal mothers who volunteered for the NCP studies were sampled due to small population sizes, summarized in Table I. Signed informed consent was obtained from each participant. The study protocols were reviewed and approved by the research ethics boards for each of the participating centres and health authorities, as appropriate.

**Table I:** Demographic variables for mothers from the Inuvik Region of the Northwest Territories, and the Baffin Region of Nunavut

	Inuvik		Baffin	
		
	Baseline study: 1998–1999	Follow-up study: 2005–2006	Baseline study: 1997	Follow-up study: 2005–2007
Age (years)				
Mean (range)	25.6 (15–45)	25.3 (16–39)	25.3 (15–39)	24.1 (15–39)
Ethnicity				
Inuit	32.6% (n=31)	68.4% (n=54)	88.6% (n=31)	100% (n=101)
Dene/Metis	44.2% (n=42)	24.1% (n=19)	N/A	N/A
Non-Aboriginal	23.2% (n=22)	7.6% (n=6)	11.4% (n=4)	N/A
Parity (No. of children)				
1	40.4% (n=38)	36.7% (n=29)	34.3% (n=12)	21.8% (n=22)
2	24.5% (n=23)	22.8% (n=18)	14.3% (n=5)	23.8% (n=24)
3	19.1% (n=18)	12.7% (n=10)	17.1% (n=6)	24.8% (n=25)
4+	16.0% (n=15)	27.8% (n=22)	34.3% (n=12)	29.7% (n=30)

### Statistical analysis

The polychlorinated biphenyl congener PCB 153 and total mercury were chosen for subsequent analysis because they were nearly 100% detected (99.04%) and they had amongst the highest variability of all chemicals examined. Here, PCB 153 is expressed on a wet weight basis in plasma (µg/L); total mercury is presented in whole blood (µg/L). Probability plots and the Anderson–Darling test demonstrated that both chemicals were lognormally distributed. Statistical inferences were performed on log-transformed data; hence geometric means were assessed on the original scale for a multiplicative effect by determining the percentage increase or decrease in chemical concentration that was detectable and significant.

We first performed a temporal analysis where a population is re-sampled in subsequent time periods, making the assumption that each time point selects an independent sample of expectant mothers. While independence is typically not assumed for time series or longitudinal studies, the type of temporal dependence is difficult to determine from only two time periods. Further, the sampling design is such that each time period may necessitate a different sample of pregnant women, since the overall population is small and pregnancy is a temporary condition that is difficult to predict. We based these calculations on powers from the analysis of variance (ANOVA) hypothesis test. We note that these calculations were approximate since we assumed the estimate of the variability (mean square error or MSE) was constant for two or more time periods. If the MSE were to change, the calculated sample sizes would be different.

We also examined a situation where 2 groups (e.g. region, ethnicity, or community) of equal size are sampled during the same time period. From the lognormal distributions, we were able to declare the geometric mean of the first group as being significantly different from the second if it is greater than the corresponding upper confidence limit, or less than the lower confidence limit. This method of calculating confidence intervals on the log scale ensures the lower bound is always positive. As a result, however, they are no longer symmetric about the geometric mean. These group sample size calculations are based on the observed variation of a single pooled dataset for each chemical, expressed as the log of the standard deviation (logsd).

Sample size calculations were based on 80% power (β=0.20) and using a significance level (α) of 0.05, at a 95% confidence interval on the log scale. All statistical analyses were performed using the software package SAS Enterprise Guide 4.2 (Statistical Analysis System).

## Results and discussion

Table II presents our temporal analysis in terms of measurable percent decreases in geometric mean concentrations for PCB 153 and total mercury, in anticipation of future decreases in human chemical concentrations. We observed that fewer samples are required as the number of time periods increases. For instance, in order to detect an overall 35% decrease in PCB 153 concentration, we would need to sample 40 mothers over each of 2 time periods, 33 mothers over each of 3 time periods, and so on. The number of samples required to measure the same percent change in total mercury is slightly higher.

**Table II:** Percent decrease in geometric mean concentrations for PCB 153 and total mercury that can be detected for different sample sizes as the number of time periods increases (MSE=0.8757 for PCB 153; MSE=1.0422 for total mercury)

Log difference	% Decrease	Time periods	Sample size per period	Time periods	Sample size per period	Time periods	Sample size per period	Time periods	Sample size per period
PCB 153									
0.10	10.5	2	345	3	283	4	240	5	210
0.15	16.2	2	154	3	127	4	108	5	94
0.20	22.1	2	87	3	72	4	61	5	54
0.30	35.0	2	40	3	33	4	28	5	25
0.40	49.2	2	23	3	19	4	16	5	15
0.50	64.9	2	15	3	13	4	11	5	10
0.60	82.2	2	11	3	9	4	8	5	7
0.70	101	2	9	3	7	4	6	5	6
0.80	123	2	7	3	6	4	5	5	5
0.90	146	2	6	3	5	4	5	5	4
1.00	172	2	5	3	5	4	4	5	4
Total mercury									
0.10	10.5	2	410	3	336	4	286	5	250
0.15	16.2	2	183	3	150	4	128	5	112
0.20	22.1	2	104	3	85	4	72	5	64
0.30	35.0	2	47	3	39	4	33	5	29
0.40	49.2	2	27	3	22	4	19	5	17
0.50	64.9	2	18	3	15	4	13	5	11
0.60	82.2	2	13	3	11	4	9	5	8
0.70	101	2	10	3	8	4	7	5	7
0.80	123	2	8	3	7	4	6	5	5
0.90	146	2	7	3	6	4	5	5	5
1.00	172	2	6	3	5	4	4	5	4

Our group analysis in Table III describes the percent change that can be measured between 2 populations during a single time period. For example, a sample size of 40 participants (for each group) allows a 39.1% decrease or a 64.3% increase in the PCB 153 concentration to be detected. In other words, if the geometric mean concentration of the first group is 1 µg/L, then the second group is significantly different if its geometric mean is smaller than 0.609 µg/L or larger than 1.643 µg/L. As before, the estimates for total mercury are slightly larger. Chemicals with less variability will have a higher degree of precision using the sample sizes presented here.

**Table III:** Percent increase and decrease in geometric mean concentrations for PCB 153 and total mercury that can be detected when comparing 2 different groups of equal size (logsd=1.132 for PCB 153; logsd=1.203 for total mercury)

	PCB 153		Total mercury	
		
Group sample size	% Decrease	% Increase	% Decrease	% Increase
10	62.9	170	65.2	187
15	55.5	125	57.7	137
20	50.4	102	52.6	111
30	43.6	77.4	45.6	83.8
40	39.1	64.3	41.0	69.4
50	35.8	55.9	37.6	60.2
60	33.3	50.0	35.0	53.8
70	31.3	45.5	32.9	49.0
80	29.6	42.0	31.1	45.2
90	28.2	39.2	29.6	42.1
100	26.9	36.9	28.4	39.6

These results demonstrate that as study population sizes are increased, smaller percent changes in chemical concentrations can be detected. However, this approach for improving method sensitivity may present a challenge for future biomonitoring in the Canadian Arctic, particularly during maternal studies, due to the inherent challenges of examining sparse populations in remote regions such as the Arctic (5). Improvements in method sensitivity may be better achieved by increasing the number of sampling time periods from 2 to 4 or 5 in order to achieve a precision of less than 20%. The northern data examined here indicates that it has been possible to confidently detect contaminant concentration decreases in the range of 20 to 50%, depending on the chemical, based on prior sample sizes ranging from 30 to 100 mothers (2). However, since these samples are not random population samples, care must be taken when making conclusions on sample size and temporal trends. We also acknowledge that with additional time points, other statistical approaches to analysing trends over time would involve regression and/or time series methods. Such methods are more computationally involved and typically require more than 5 time periods to adequately assess the changes in contaminant levels over time.
